# Progress in Surgery

**Published:** 1901-10-12

**Authors:** 


### Progress in Surgery.

### GENITO-URINARY DISEASES.

**Acute Gonorrhœa.**—Gerald Dalton [1] has written on the treatment of this affection. He points out that it is generally accepted that if gonorrhœa lasts more than six weeks the posterior (membrano-prostatic) urethra becomes invaded. It is almost impossible for patients themselves to flush out this portion of the urethra. Dalton outlines the technique of the treatment of gonorrhœa by urethral irrigation based on the methods described first by Janet and later by Valentine. The abortive treatment may be tried when on the first or second day of the attack there is a tickling or burning sensation in the urethra, and a mucoid secretion containing epithelial cells and gonococci, but no pus cells. The treatment may be very painful and may fail to cure the disease. After urinating the urethra is irrigated through a No. 6 English catheter passed in four inches. Then the walls of the urethra are swabbed through a urethroscopic tube with protargol solution, 50 grs. to 1 oz., beginning at a distance of 4 inches from the meatus. In a few hours there may be purulent discharge. If successful the discharge disappears in four or five days. Astringents may be used for a day or two to dry the mucous membrane. In the more ordinary treatment rest is very important. The patient is to ride rather than walk, to sit rather than stand, and to avoid horseback riding, cycling, dancing and rowing. The testicles should be supported. Saline cathartics are to be avoided. Magnesium sulphate irritates the urethra. A cathartic pill every third day is better.

Potass. bicarb. and acetate combined with tinct. hyoscy. should be given. The following may be used as an injection for the urethra just before retiring and retained five minutes, to prevent chordee :—viz., Liq. morph. hydrochlor., 15 minims ; cocaine hydrochlor., ½ gr. ; Aq. ad 2 drms. If irrigation is decided upon, the earlier it is begun the better. It is not unusual to get the cases completely well in three weeks. Valentine's very convenient irrigation apparatus is employed, consisting of a receiver, which travels up and down a slide fixed against the wall, provided with tubing, stop-cock, shield, and glass nozzle. The fluid is used hot. When the stop-cock is only partially turned on, and the nozzle only loosely inserted into the meatus, only the anterior urethra is flushed. By turning the stop-cock fully on and pressing the nozzle firmly into the meatus the fluid will pass the compressor muscle and get into the bladder. To help this, the patient must take a deep breath and make an effort to pass water. The fluid is finally expelled from the bladder by natural efforts. During posterior irrigation no solution at all should pass out of the meatus. If seen early, and the irrigation treatment carried out once, or better twice, daily, at the end of a fortnight there will probably be only a remaining discharge of a few drops of clear or milky fluid. An astringent injection is then better than irrigation. The injection fluid should be retained in the urethra one to four minutes and used three or four times daily for a week. Permanganate of potash solution has been chiefly employed for the irrigation, but Dalton is convinced that protargol is the most useful drug. He begins in acute cases with a strength of 1 to 400. By the fifth day 1 to 200 may be used, which strength is in many cases sufficient, though a 1 in 100 solution is sometimes advisable. Ichthyol irrigations of 2 per cent. strength may be used to finish up with. They seem to have a sedative and tonic effect.

**Chronic Gonorrhœa or Gleet.**—The technique of irrigation is the same as above, but most of the fluid is to be used for the posterior urethra, the bladder being filled several times. The other methods of treatment are (1) by the direct application of strong solutions to the diseased areas through the canula of the urethroscope ; or (2) by instillation, *e.g.*, with Guyon's syringe ; or (3) by the use of the cold sound or psychrophor ; or (4) the use of antrophores, which consist of small coiled springs, coated with an insoluble substance, outside of which is a soluble mass containing various drugs. The antrophores are 6 or 9 inches long, and are introduced on going to bed and left in 15 minutes. As an irrigating fluid protargol is recommended at first, to be followed by one of zinc sulphate and alum finally. F. C. Valentine[2] points out that gonorrhœa may become chronic from misdirected treatment, over-treatment, lack of treatment, and from congenital or acquired deformities and complications. Hot external applications constantly applied will generally relieve preputial infiltration. If not, and if it remains impossible to retract the prepuce, it is best to make a free incision through each side, exactly midway between the dorsal line and the frænum. A stenosed meatus generally requires meatotomy. Stenosis of the posterior boundary of the fossa navicularis may exist with a small meatus or independently, and requires similar treatment. Minute para-meatal fistula may harbour the gonococci, and require division. Warts may prolong gonorrhœa. The pedunculated ones almost always yield to the application of powdered savin. If that fails, they may be painted with tincture of iron and curetted daily. Residua of previous infections of the urethra, however long quiescent, can at any time produce an attack resembling that of a new acute gonorrhœa. The most important residua are infiltration of the mucosa, stricture, retention of gonococci in the crypts, glands and follicles, and neoplasms. The fact that a large-sized sound glides easily through the urethra is not a proof that there is no obstruction. In such cases a soft bougie-à-boule may be obstructed by a series of contractures when withdrawn. The urethroscope is necessary for precise diagnosis. Valentine's urethroscope carries the light inside the urethroscopic tube so that the source of light is close to the spot to be illuminated. The instrument enables one to distinguish anæmia and hyperæmia of the mucosa ; soft, medium, and hard infiltrations, epithelial denudations, strictures, infiltrations of the crypts, glands, and follicles, intra-urethral chancre, chancroid, benign and malignant growths. The denudations are treated with nitrate of silver in 1 or 2 per cent. solutions. Infiltrations and strictures are best treated by gradual dilatation. For this, while the urethral calibre is below 21, Charrière, or hyperæsthesia exists, soft conical sounds are used.

The use of cocaine is deprecated. It is said to render urination more persistently painful afterwards. In the next stage Oberlaender's and Kollmann's various dilators are employed and the dilatations performed exactly in the affected regions. These instruments are covered with fine, sterilisable rubber covers. The above treatment often suffices to cure also infiltrations of the urethral crypts, glands and follicles. If not these affections must be attacked locally through the urethroscope with special instruments. Valentine advocates the use of synol soap for disinfecting the hands and instruments and as a lubricant ; also that the patients themselves should use it for their hands. The writer then considers the subject of marital re-infection. Both men and women may harbour gonococci in a latent manner for years, and then, as it were, re-infect one another, causing acute symptoms, though there has been no infidelity.

**Protargol in Gonorrhœa.**—Niessen[3] has published some results of the treatment of gonorrhœa with protargol. He has used it in 244 cases in soldiers. Ninety-nine of these cases were uncomplicated ones of anterior urethritis, and it is these that are analysed. The patients were kept in bed 10 to 14 days. In 34 cases complications developed during the treatment. In these cases 13 had bladder symptoms, five inflammation of the testes, and 29 urethritis posterior. The drug was never used in greater strength than ¾ per cent. The average duration of treatment was 39·7 days. Niessen concludes that solutions of the above strength produce no discomfort, that in the gleety stage astringents work better, and that the disease is not cut short by the drug.

[1] Edin. Med. Journ., July, 1901. [2] Med. Rec., June, 1901.

[3] Brit. Med. Journ., Epit., May, 1901.

